# Unique local gradient-based functional fingerprints enable precisely identifying individual infants over ages

**DOI:** 10.1016/j.dcn.2026.101828

**Published:** 2026-10-03

**Authors:** Xinrui Yuan, Dan Hu, Jiale Cheng, Zhengwang Wu, Li Wang, Weili Lin, Gang Li

**Affiliations:** Department of Radiology and Biomedical Research Imaging Center, University of North Carolina at Chapel Hill, Chapel Hill, 27599, NC, USA

**Keywords:** Local gradient map, Functional fingerprints, Infant brain, Cognition

## Abstract

Brain functional connectivity (FC) exhibits individualized patterns that can serve as reliable "fingerprints" for identifying individual adults. However, the rapid and complex development of the infant brain poses significant challenges in establishing individualized, developmentally-robust functional fingerprints during infancy. In this study, we introduce a novel approach that captures fine-grained local gradients of FC derived from functional MRI, enabling precise characterization of unique subject-specific FC patterns. Our method achieved approximately 99% individual identification accuracy across longitudinal scans spanning infancy in 103 infants, substantially outperforming conventional connectome-based fingerprinting, which achieved approximately 70% accuracy. Importantly, these local gradient-based functional fingerprints, predominantly localized within association and sensory networks, were also modestly but significantly associated with infant cognitive performance. Together, these findings support the existence of individualized functional fingerprints that are stable across dynamic early brain development, offering new insights into the early development of functional individuality and its cognitive relevance.

## 1 Introduction

Resting-state functional MRI (rs-fMRI) is a powerful, noninvasive technique for mapping brain functional connectivity (FC) and architecture in vivo. In adolescence and adulthood, it has been observed that individuals exhibit highly stable, unique, individualized FC patterns, i.e., functional “fingerprints”, that enable reliable identification of individuals over ages (Emily et al., 2015, Horien et al., 2019, Miranda-Dominguez et al., 2018, Demeter et al., 2020, Kaufmann et al., 2017, Jalbrzikowski et al., 2020). In contrast, FC in infancy shows significantly lower identifiability and stability, likely reflecting the dynamic, complex neurodevelopmental processes occurring during early life, which influences both brain structure (Gilmore et al., 2018, Li et al., 2014) and function (J Paterson et al., 2006, Graham et al., 2015, Zhang et al., 2019a, Gao et al., 2015a). These dynamic developmental changes introduce greater variability in functional patterns, making infant functional connectomes inherently less stable than those of older individuals. Given that infancy is a critical window for shaping later cognitive and behavioral performance (Kaufmann et al., 2017), it is essential to precisely characterize and identify stable individualized functional fingerprints during this period. Moreover, linking these early individualized functional fingerprints to behavioral phenotypes could inform personalized, developmentally sensitive interventions aimed at optimizing long-term outcomes.

Conventionally, functional fingerprinting is achieved by comparison of FC matrices encoding inter-regional connection strengths between a set of regions-of-interest (ROIs) from predefined brain atlases, such as those containing 268 (Emily et al., 2015), 333 (Gordon et al., 2016), 602 (Hu et al., 2022) or over 1000 ROIs (Kong et al., 2021), which are widely applied to characterize complex functional patterns in both matured and developing brains. Despite employing various brain parcellations, connectome-based individual identification accuracies over ages during infancy persistently remain low, e.g., only around 70% (Hu et al., 2022). This is likely because the neurodevelopment is governed by dynamic processes with the enhancement of functional segregation and integration during infancy (Wen et al., 2019, Gao et al., 2015b). While connectome-based FC describes ROI-scale information, it cannot well capture the fine-grained, spatially localized detailed patterns, which could be more robust and developmentally invariant to define early functional fingerprints. Moreover, predefined brain parcellation schemes impose inherent constraints on fMRI analysis during infancy, as this not only limits the spatial resolution of analysis, but also makes a problematic assumption that the parcellation should be unchanged during dynamic early brain development. These limitations underscore the necessity to develop advanced approaches capable of capturing intricate fine-grained functional patterns that can precisely characterize the individualized function fingerprints throughout infancy.

Local gradients of FC have recently emerged as a powerful approach for characterizing cortical functional organization (Schaefer et al., 2018, Wig et al., 2014, Cohen et al., 2008). By detecting abrupt spatial transitions in connectivity patterns, local gradients delineate fine-scale functional boundaries and have been widely used for parcellation (Gordon et al., 2016, Schaefer et al., 2018, Wig et al., 2014, Bijsterbosch et al., 2018, Kong et al., 2019, Tu et al., 2025). These fine boundaries are highly reproducible across sessions (Schaefer et al., 2018) and have been indicative for neighboring regions with distinct neuronal subtypes and gene expression profiles (Hawrylycz et al., 2012), indicating that local gradients capture biologically meaningful, detailed individualized information that is potentially useful for functional fingerprinting.

In this work, for the first time, we leverage the local gradient maps (LGMs) to characterize individualized functional characteristics to enable precisely identifying individual infants over ages despite the dynamic, complex early brain development, without reliance on any brain parcellations. Specifically, our aims were to: (i) determine whether LGMs can capture individualized, developmentally-robust functional characteristics during infancy, thus enabling individual identification over ages; (ii) identify which cortical regions manifest the highest individualized identifiability and uniqueness during infancy in terms of LGMs; and (iii) examine the association between LGM-based functional fingerprint and early cognitive performance. Addressing these questions holds great significance in neuroscience, yielding new perspectives on developmentally-robust, individualized intrinsic patterns of functional organization and their cognitive relevance.

## 2 Results

Our functional fingerprinting and cognition analyses were performed on 132 individuals from the public Baby Connectome Project (BCP) dataset (Gilmore et al., 2018) aged from 16 to 874 days. Detailed inclusion and exclusion of this study is presented in Fig. 1**a**. The fingerprinting cohort, comprising 103 subjects with longitudinal scans, was stratified into three distinct datasets (I, II, and III), characterized by varying age distributions and session gaps (the time interval between two fMRI sessions). Within each dataset, each subject contributed two longitudinal scans at different ages with session 1 (S1) earlier than session 2 (S2). Specifically, Datasets I, II, and III are comprised of the first two sessions, the last two sessions, and the first and last sessions of each individual, with the mean session gaps of 147, 191, and 298 days, respectively. Detailed demographic information of these datasets is presented in Fig. 2.

**Fig. 1:**
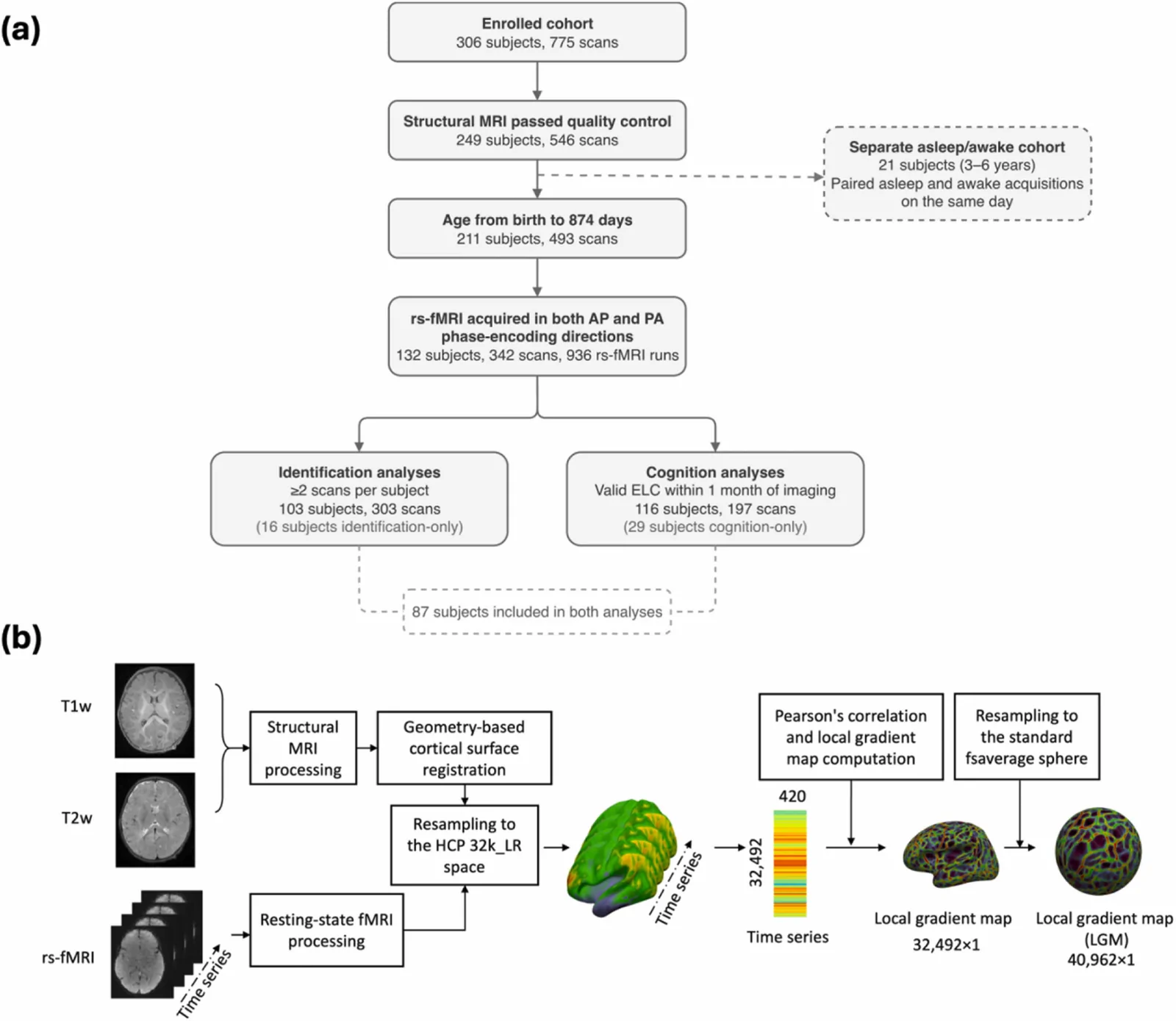
(a) Participant flow and sample sizes at each inclusion stage. A scan denotes a single imaging timepoint (one visit, anchored on the T1w or T2w acquisition), a run denotes an individual rs-fMRI acquisition within a scan. Of the 132 subjects in the analytic pool, 87 were included in both the identification and cognition analyses, 16 were included only in the identification analyses because they had no eligible ELC-matched scan, and 29 were included only in the cognitive analyses because they did not meet the longitudinal scan requirement for identification. The cross-state cohort underwent the same quality-control procedure but fell outside the specified age window and was therefore not included in the counts shown above. (b) An illustration of data processing. Detailed steps are described in the following corresponding parts as: structural and functional MRI processing (Sections ‘Structural MRI processing’, ‘Functional MRI processing’, and ‘Cortical surface reconstruction and mapping’) and computing local gradient map of functional connectivity for each scan (Section ‘Computation of local gradient map (LGM) of functional connectivity’).

**Fig. 2:**
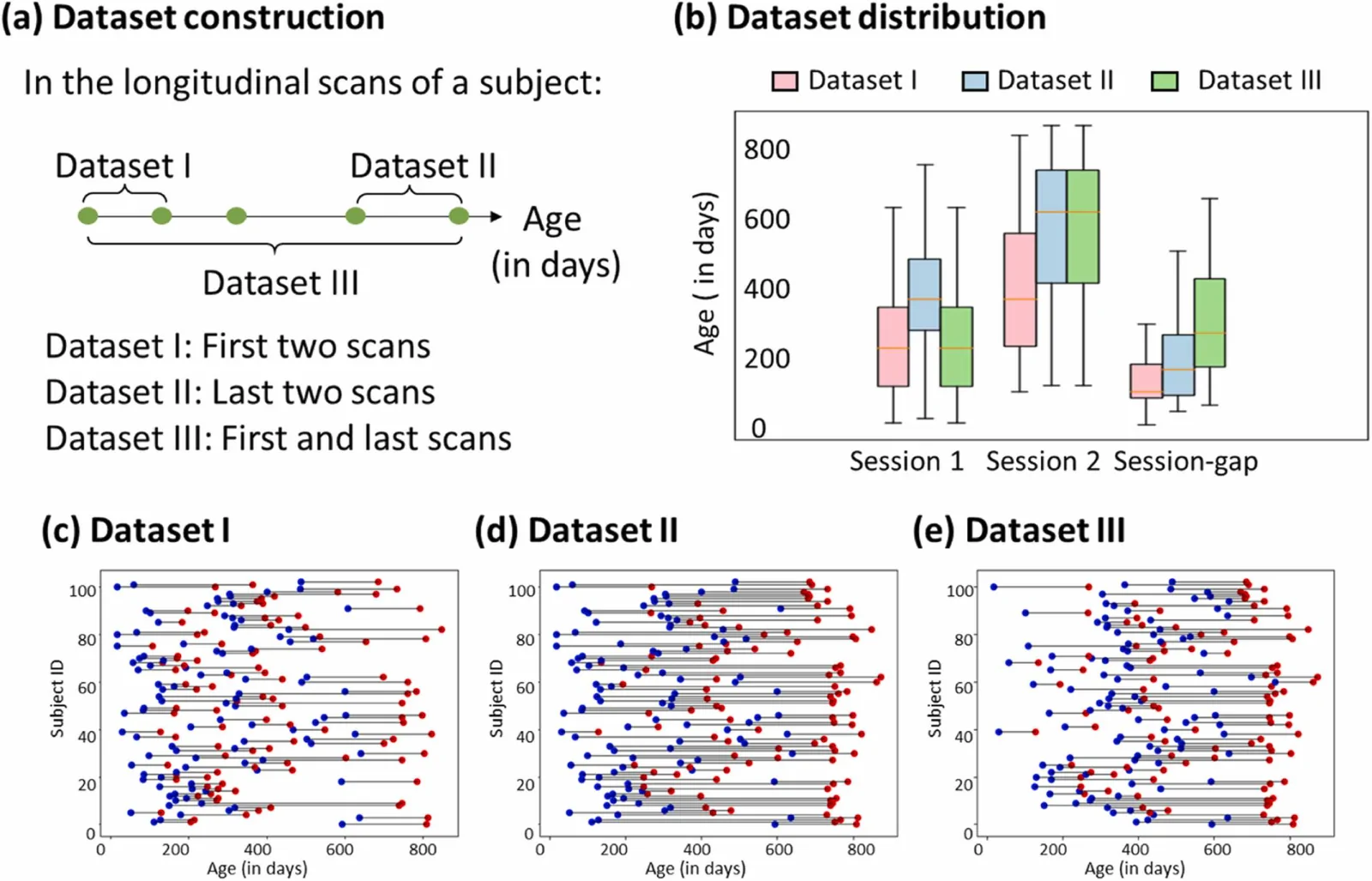
Dataset description. (a) Dataset construction. To study the functional fingerprints under different distributions of ages and session gaps, Datasets I, II, and III consist of the first two scans, the last two scans, and the first and last scans for each subject, respectively. (b) The detailed age distribution information of the three datasets, the box plots of the scan ages of session 1 and session 2, and their session gaps (age difference between two sessions). (c)-(e) The scan distribution of Datasets I–III, where the blue and red points represent the scans acquired from session 1 and session 2, respectively.

Individual identification was performed between a target set and a base set. Two longitudinal scans from the same subject were assigned into the base set and the target set, respectively. Given a subject in the target set, for each cortical vertex in the local gradient maps (LGMs), we computed its local similarity with each subject’ LGM in the base set based on the correlation of local patterns in a surface patch, resulting in one local correlation map (LCM) per target-base comparison. Identity was predicted using a vertex-wise majority-voting approach, where each cortical vertex identifies an individual with the highest local similarity from the base set, and the final predicted identity was then determined by majority vote across all vertices. Since fingerprinting was performed based on local patches, this procedure is referred to as local-level LGM-based fingerprinting (LGM-Local). Unless otherwise specified, we omit this notation for simplicity. The identification rate was defined as the percentage of subjects whose identities were correctly recognized out of the total number of subjects (see **Methods**).

### 2.1 Local gradient map-based functional fingerprinting

**Whole-brain-based identification.** First, LGM-based individual identification was performed using all vertices from both hemispheres (81,924 vertices in total), with S1 serving as the target session and S2 as the base session (S1→S2), and with their roles reversed (S2→S1). Within each outer fold of the subject-level nested cross-validation, the patch number and patch size were selected exclusively using the corresponding inner folds and were then applied to the held-out outer test subjects. The resulting identification accuracies were 100.0 ± 0.0%, 99.1 ± 2.7%, and 98.2 ± 3.6% for Datasets I, II, and III, respectively, in the S1→S2 direction. The reverse S2→S1 direction yielded comparable accuracies of 100.0 ± 0.0%, 100.0 ± 0.0%, and 99.0 ± 3.0%, respectively (mean ± SD across outer folds, Fig. 3**a**). These nested cross-validation results constitute our primary estimates of identification performance.

**Fig. 3:**
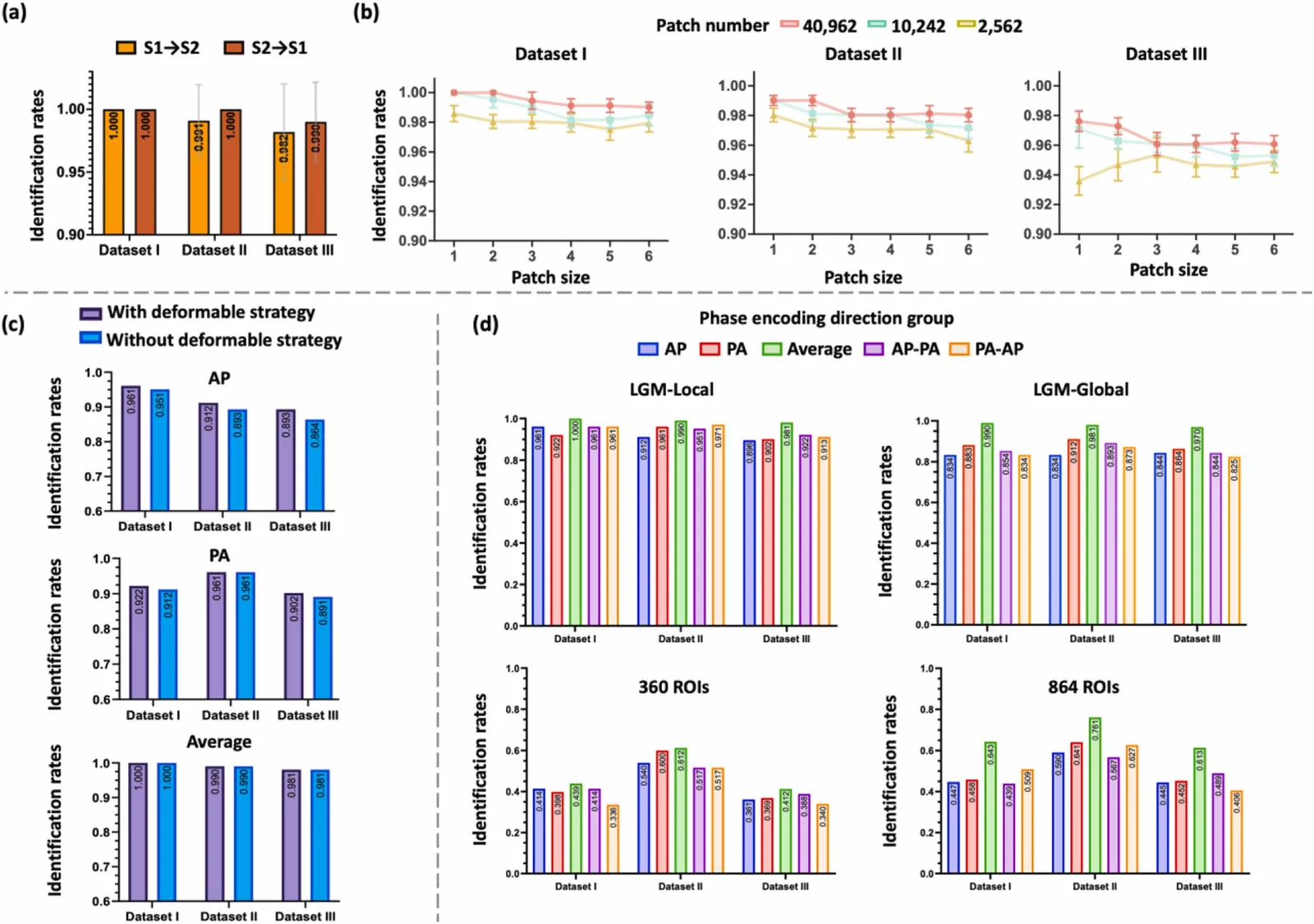
(a) Held-out identification accuracy under subject-level nested cross-validation, evaluated in both the S1→S2 and S2→S1 directions. Within each of the 10 outer folds, patch number and patch size were selected using the corresponding inner folds and then applied to the held-out outer-fold subjects. Results are shown as the mean ± SD across outer folds. (b) Inner-cross-validation accuracy across 18 combinations of patch number (40,962, 10,242, and 2562 patches per hemisphere) and patch size (r∈{1, 2, 3, 4, 5, 6}). Results are shown as the mean ± SD across the 10 outer folds. Based on its overall accuracy and stability, 40,962 patches with patch size r = 1 was selected as the fixed configuration for subsequent descriptive analyses. (c) Full-sample identification rates for all 103 subjects in each dataset, with and without the deformable strategy, using the fixed configuration selected from the inner-cross-validation results in (b). (d) Full-sample identification rates for all 103 subjects in each dataset across different phase encoding directions via local-level and global-level LGM-based functional fingerprinting (LGM-Local and LGM-Global), and comparison with connectome-based approaches (360 ROIs (Glasser et al., 2016) and 864 ROIs (Wang et al., 2023a)). Panels (c) and (d) are descriptive analyses, and no additional parameter selection was performed.

After completing the nested cross-validation analysis, we selected 40,962 patch centers per hemisphere (81,924 in total) with a patch size of *r* = 1 as a common configuration for the full-sample and subsequent descriptive analyses, based on the inner-cross-validation results described below. Using this fixed configuration, the full-sample identification rates were 103/103 (100.0%), 102/103 (99.0%), and 101/103 (98.1%) for Datasets I–III in the S1→S2 direction, with identical results in the S2→S1 direction. Since the S1→S2 and S2→S1 directions produced same results, subsequent analyses focus on S1→S2 unless otherwise specified. These full-sample results are provided as descriptive summaries and do not replace the held-out nested cross-validation estimates reported above.

Permutation testing of the full-sample results further showed that identification performance was significantly above chance. The null distributions were centered near the theoretical chance level of 1/103, and none of the 5000 permuted accuracies reached the corresponding observed accuracy in either direction for any of the three datasets (empirical *p* = 1/5001 ≈ 0.0002 for each comparison).

To further distinguish longitudinal identifiability from developmental invariance, we examined within-subject fingerprint similarity in relation to both inter-scan age gap and developmental age. LGM fingerprints not only remained individually distinctive across longitudinal scans but also showed variation in within-subject similarity across inter-scan intervals and developmental stages (**Supplementary Results**, Figure S1).

### 2.2 Factors affecting identification accuracy

**Identification using different patch sizes and patch numbers.** We conducted this experiment to investigate the impact of the resolution of LCMs on their ability to characterize the individualized infant functional characteristics. The computation of LCMs is affected by two key variables: the patch number (the number of sampled cortical vertices) and the patch size (defined by *r*-ring neighborhood on the cortical surface). We first uniformly sampled vertices on LGMs, followed by the selection of varying patch sizes centered on each sampled vertex. The patch size determines the spatial extent of local information incorporated into the similarity computation between LGMs, with larger patch sizes containing more extensive local topological information.

To evaluate the effects of these parameters without compromising the separation between model selection and performance estimation, we used inner cross-validation accuracy rather than accuracy calculated from the full sample. This is the same criterion used to select the fixed configuration for the primary analyses. We evaluated all combinations of patch number within {40,962, 10,242, 2562} and patch size (*r* ∈ {1, 2, 3, 4, 5, 6}). Accuracy was first averaged across the five inner folds within each outer fold and is reported as the mean ± SD across the 10 outer folds (Fig. 3**b**).

Overall, the configuration with 40,962 patches and a 1-ring neighborhood produced the highest and most stable inner cross-validation accuracy. At this resolution, performance varied little across patch sizes, remaining approximately 99–100% in Dataset I, 98–99% in Dataset II, and 96–98% in Dataset III.

At the intermediate resolution of 10,242 patches, identification accuracy showed a modest decline as patch size increased. From *r* = 1 to *r* = 6, accuracy decreased from approximately 100–98.5% in Dataset I, from 99% to 97% in Dataset II, and from 97% to 95% in Dataset III. At the lowest resolution of 2562 patches, Datasets I and II remained relatively stable, with accuracy ranging from approximately 96–99%. Dataset III showed a slightly different pattern, with accuracy increasing from approximately 94% at *r* = 1 to approximately 95% at larger patch sizes.

These findings indicate that using more densely sampled patches generally improves identification accuracy and reduces its sensitivity to patch size. The effect of patch size was most apparent at the intermediate resolution, where smaller neighborhoods performed modestly better. At the lowest resolution, its effect was dataset-dependent, whereas at the highest resolution, performance was largely insensitive to the neighborhood size examined. The consistently strong performance of the 40,962-patch, 1-ring configuration therefore supports its use as the fixed setting for the primary held-out evaluation (Fig. 3**a**) and the subsequent descriptive analyses (Fig. 3**c, d**).

**Identification across different phase encoding directions.** Both anterior-to-posterior (AP) and posterior-to-anterior (PA) phase encoding directions (PEDs) have been used to acquire rs-fMRI. To investigate the potential influence of phase encoding directions on functional fingerprinting, we systematically evaluated the following five experimental configurations: (1) both target and base sets comprising AP scans exclusively (AP group); (2) both target and base sets comprising PA scans exclusively (PA group); (3) target set comprising AP scans while base set comprising PA scans (AP-PA group); (4) target set comprising PA scans while base set comprising AP scans (PA-AP group); and (5) both target and base sets comprising averaged AP and PA scans (Average group).

The LGM-based functional fingerprinting demonstrated robust and consistent performance when target and base sets shared the same phase encoding direction (Fig. 3**d**). For the AP group, identification rates on Datasets I, II, and III were 96.1%, 91.2%, and 89.3%, respectively. Comparable performance was observed in the PA group, with rates of 92.2%, 96.1%, and 90.2%. Notably, the average group exhibited superior performance, achieving identification rates of 100%, 99%, and 98.1% across the three datasets.

When analyzing configurations across phase encoding directions (AP-PA and PA-AP groups), the identification rates maintained relative stability. The AP-PA group achieved rates of 96.1%, 95.1%, and 92.2% on Datasets I, II, and III, respectively, while the PA-AP group demonstrated rates of 96.1%, 97.1%, and 91.3%. These results suggest that the LGM-based approach maintains robust performance even across different phase encoding directions. Of note, these results were reported on the full 103-subject sample since we use the fixed configuration selected by the nested cross-validation above (40,962 vertices, *r* = 1) and introduce no further configuration selection.

**Identification using deformable strategy.** To account for potential registration errors and enhance robustness under single phase-encoding scans, we developed a deformable strategy in the LCM computation (see **Methods**), incorporating patch rotation (±60°) and translation (six directional axes) on the spherical surface. We repeated vertex-wise identification with this strategy (Fig. 3**c**).

The deformable strategy improved identification for single phase-encoding scans. In the AP group, rates increased from 95.1%, 89.3%, and 86.4–96.1%, 91.2%, and 89.3% across Datasets I–III, and in the PA group from 91.2%, 96.1%, and 89.1–92.2%, 96.1%, and 90.2%, indicating that it mitigates local patch misalignment and reduces interference from signal noise and preprocessing artifacts under noisier single-direction acquisitions.

On the averaged scans, however, the deformable strategy yielded no further gain (identical rates of 100%, 99%, and 98.1% with and without it), because averaging AP and PA scans already suppresses the noise that the deformable strategy is designed to correct. Since our main analyses use averaged scans, this correction is unnecessary there. The deformable strategy is therefore not part of the main identification pipeline or the configuration search and is reported only as a robustness analysis for single phase-encoding acquisitions.

**Comparison with connectome-based fingerprinting.** To demonstrate the advantages of our LGM-based functional fingerprinting framework, we conducted a comparative analysis against conventional connectome-based fingerprinting methods under different phase-encoding directions. A potential concern in such a comparison is that any observed advantage might arise from the identification procedure rather than from the underlying representation. To separate these two factors, we varied the representation (connectome vs. LGM) and the identification procedure (global vs. local) independently. Our main experiments used a local-level procedure (LGM-Local), which captures fine-grained spatial similarity patterns across local cortical patches. We additionally evaluated a global-level variant (LGM-Global), which applies exactly the same decision rule as connectome-based fingerprinting to the LGM representation, and therefore serves as a procedure-matched control. Therefore, the comparison between connectome-based fingerprinting and LGM-Global helps assess the contribution of the underlying representation while holding the final identification rule constant, whereas the comparison between LGM-Global and LGM-Local evaluates the additional contribution of local aggregation. Functional connectomes were constructed using two representative functional parcellations (Wang et al., 2023a, Glasser et al., 2016), comprising 360 and 864 regions of interest (ROIs), respectively, in order to control for the possibility that any advantage reflects parcellation. All LGM analyses used the configuration fixed by the nested cross-validation with no parameter re-selected for these comparisons.

Connectome-based fingerprinting analysis was also implemented across different phase encoding directions (Fig. 3**d**). In the AP group, utilizing 360 ROIs yielded identification rates of 41.4%, 54%, and 36.1% for Datasets I, II, and III, respectively. Implementation with 864 ROIs demonstrated enhanced identification rates of 44.7%, 59%, and 44.5% on the three datasets. For the PA group, identification rates using 360 ROIs were 39.8%, 60%, and 36.9% on Datasets I, II, and III, respectively, while the 864-ROI parcellation yielded improved rates of 45.8%, 64.1%, and 45.2% on the same three datasets. The average group exhibited identification rates of 43.9%, 61.2%, and 41.2% with 360-ROI parcellation, and higher rates of 64.3%, 76.1%, and 61.3% with 864-ROI parcellation on the three datasets.

LGM-global was also implemented across the 5 phase encoding directions. In the AP group, global-based LGM demonstrated identification rates of 83.4%, 83.4%, and 84.4% for Datasets I, II, and III, respectively. For PA group, 88.3%, 91.2%, and 86.4% for Datasets I, II, and III, respectively. The average group exhibited identification rates of 99.0%, 98.1%, and 97.1% for Datasets I, II, and III, respectively.

In the AP-PA group, identification rates were 41.4%, 51.7%, and 39.8% using the 360-ROI parcellation, while the 864-ROI parcellation achieved rates of 43.9%, 55.7%, and 48.9% on Datasets I–III. Similarly, the PA-AP group demonstrated identification rates of 33.6%, 51.7%, and 34.0% with 360 ROIs, and 50.9%, 62.7%, and 40.6% with 864 ROIs across the three datasets. By contrast, LGM-global fingerprinting substantially outperformed conventional connectome-based approaches, achieving identification accuracies of 85.4%, 89.3%, and 84.4% for the AP-PA group, and 83.4%, 87.3%, and 82.5% for the PA-AP group on Datasets I–III, respectively. As for the LGM-local fingerprinting, the AP-PA group achieved rates of 96.1%, 95.1%, and 92.2% on Datasets I, II, and III, respectively, while the PA-AP group demonstrated rates of 96.1%, 97.1%, and 91.3%. These results highlight the superiority of LGM-based fingerprinting in capturing subject-specific functional signatures across varying phase-encoding conditions, regardless of whether a global-level or local-level identification procedure is employed.

### 2.3 Quantifying vertex-wise contributions to identification

To quantify how different vertices contribute to fingerprinting and capture the most individualized functional characteristics, we derived two measures: uniqueness (U) and differential power (DP) (Emily et al., 2015, Wang et al., 2021a, Tian et al., 2021, Mueller et al., 2013). Uniqueness is high for vertices exhibiting large inter-subject variability while maintaining high intra-subject consistency across MRI sessions. DP, on the other hand, reflects each vertex's ability to distinguish an individual from others. Vertices with high DP values demonstrate positive contribution to individual identification. In other words, vertices with high DP values are thought to make key contributions to individual identification (see **Methods**).

We computed average vertex-wise DP and U values across three datasets and then aggregated them to 864 ROIs and further to 10 networks (Wang et al., 2023a) to identify networks contributing significantly to individual identification (Fig. 4). Our analysis revealed that vertices with high uniqueness values were predominantly located in the occipital lobe, parietal lobe, and posterior cingulate cortex, corresponding to the central visual, posterior frontoparietal, and posterior default mode networks, which exhibited strong within-subject consistency across developmental stages while simultaneously showing variability between individuals. Similarly, vertices with high DP values were concentrated within the occipital lobe, underscoring the central visual network as a key discriminative contributor to individual identification. To further characterize network contribution, we calculated (1) the within-network proportion, the percentage of vertices within each network that fell into the top 5% of all values; and (2) the between-network proportion, the percentage of the total top 5% vertices contributed by each network, for both DP and U. The results showed that most top-ranking vertices for both U and DP were concentrated within the central visual, posterior default mode, and posterior frontoparietal networks, highlighting their predominant roles in driving individual identification.

**Fig. 4:**
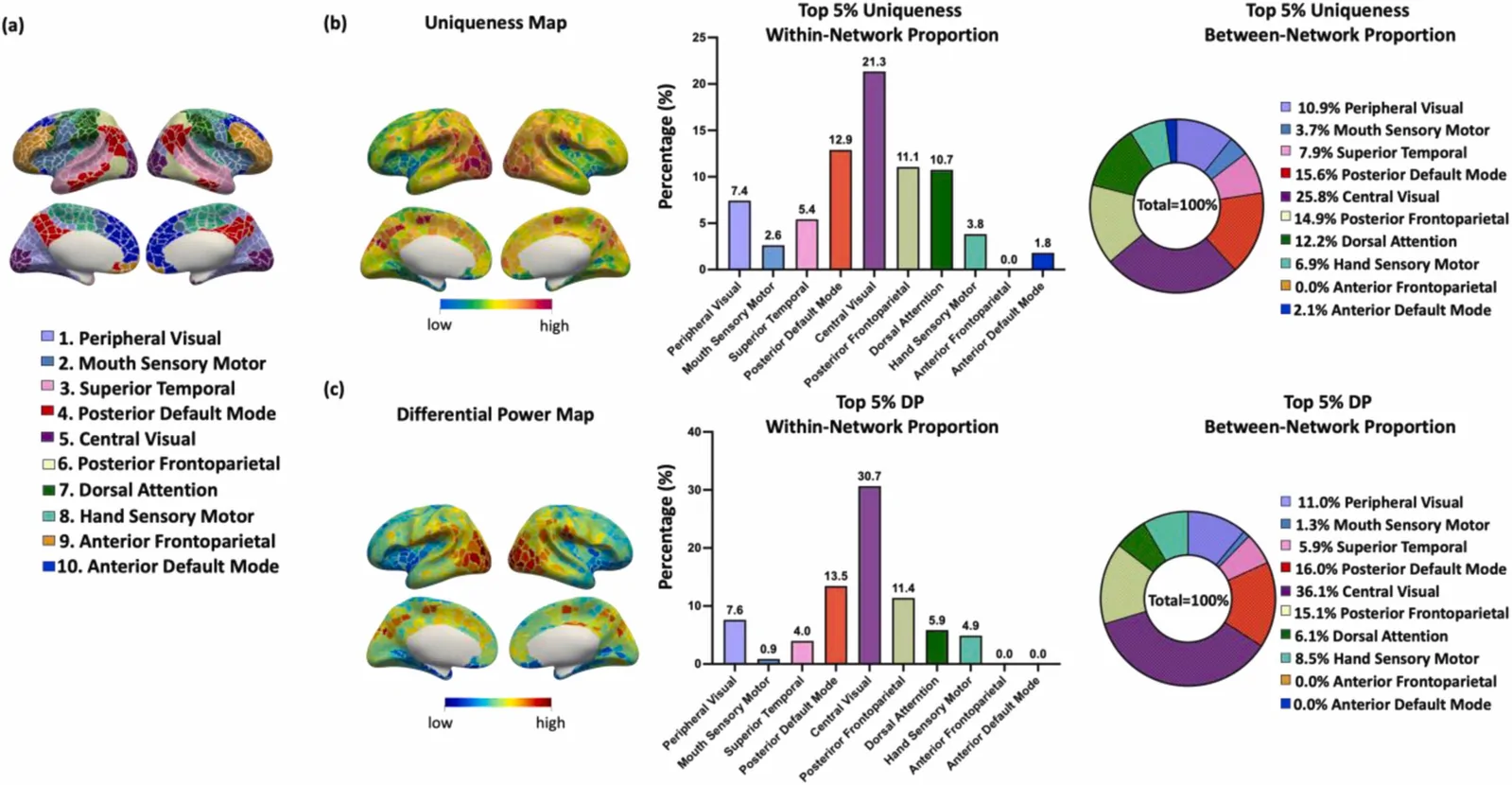
(a) Functional network definition. We used an infant-specific fine-grained functional parcellation map comprising 864 cortical regions-of-interest (ROIs) organized into ten networks, defined at 12 months of age (Wang et al., 2023a). Spatial distributions of (b) uniqueness and (c) DP values. Each row displays three panels from left to right: parcel-level average maps across three datasets, the within-network proportion (the percentage of vertices within each network that fall into the top 5% of all values), and the between-network proportion (the percentage of the total top 5% vertices contributed by each network).

### 2.4 Identification across Asleep and Awake States

To investigate whether LGMs can capture state-invariant individualized characteristics between awake and asleep states, we analyzed 21 BCP subjects (3–6 years of age) with paired asleep and awake scans on the same day. After computing LGMs, we performed individual identification using the vertex-wise approach, achieving 100% identification accuracy.

Additionally, we performed connectome-based identification using an 864-ROI parcellation (Wang et al., 2023a) to compare awake and asleep states, as finer-grained parcellations have demonstrated superior identification performance in previous studies. This analysis yielded identification rates of 66.7% for sleep-to-awake matching and 29.5% for awake-to-asleep matching, revealing an asymmetric pattern in cross-state identification. This demonstrated the superior robustness of LGMs for individual identification across asleep and awake states compared to FC.

### 2.5 Cognitive Score Prediction

To examine the behavioral relevance of local gradients of functional connectivity (FC), we predicted infant cognitive performance measured by early learning composite (ELC) (M Mullen et al., 1995, Yitzhak et al., 2015), in 116 infants (197 scans) with valid ELC assessments. Following prior evidence that network topography is behaviorally meaningful (Bijsterbosch et al., 2018, Kong et al., 2019), we specifically focused on functional boundaries, which represent interfaces of sharp connectivity transitions between adjacent cortical ROIs in the cortical parcellation (Wang et al., 2023a). These boundaries typically correspond to high local gradient values and delineate zones of functional parcellation across the cortex. The local gradient magnitudes at these boundaries were used as predictive features in the repeated subject-level cross-validation (see **Methods**). The averaged predicted and observed ELC scores showed a modest but statistically significant correlation (*r* = 0.294, 95% CI: [0.188, 0.423], *p* < 0.0001) (Fig. 5**a**). This association remained significant after additionally controlling for age at scan (partial *r* = 0.290, 95% CI: [0.187, 0.411], *p* < 0.0001). Because ELC scores are already age-normalized, residual age effects in the outcome are expected to be small, and the close agreement between the adjusted and unadjusted estimates confirms that the predictive association was not driven by age-related differences in cognitive performance.

**Fig. 5:**
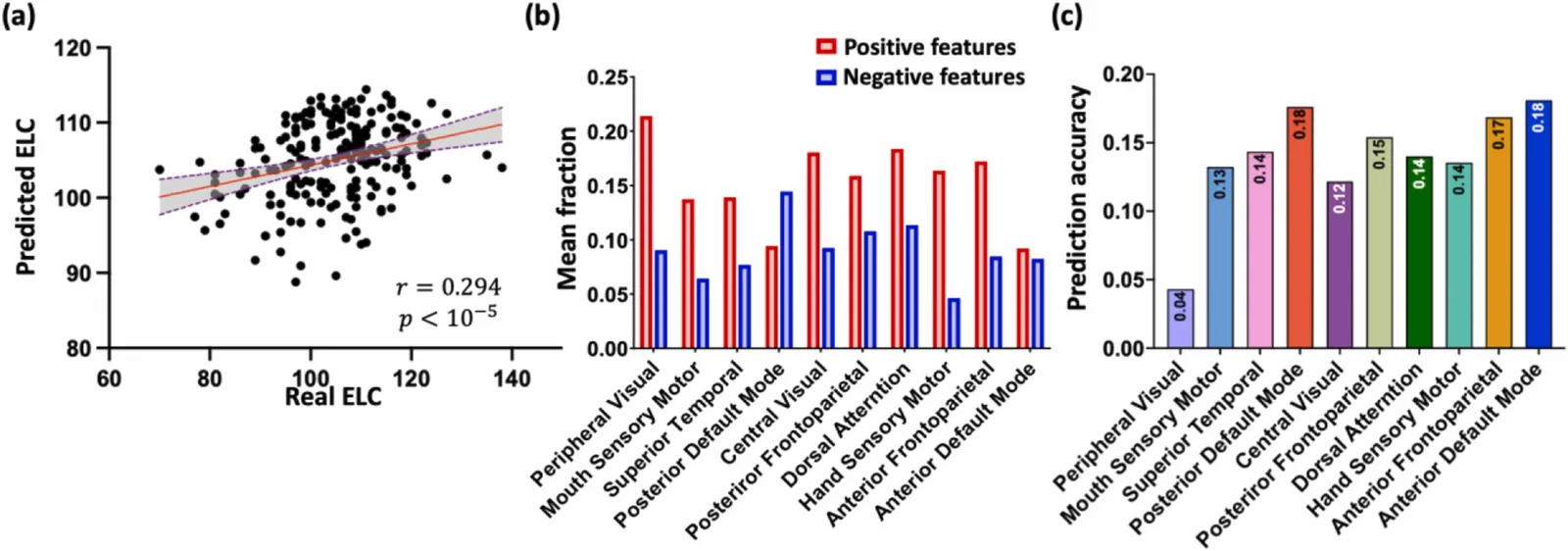
Cognitive score prediction based on local gradient maps (LGMs). (a) Scatter plots of the predicted ELC and the real ELC. Each dot represents the predicted and real ELC pair from one subject; the gray area indicates 95% confidence interval for the fit line. (b) Mean fraction of positively and negatively associated boundaries across cross-validation folds, separated by positive (red) and negative (blue) associations with ELC, and normalized by network size (i.e., the total number of boundaries within each network). (c) The prediction accuracy (r-value, Pearson’s correlation between predicted and real ELC scores) of leave-one-network-out strategy. A lower value indicates a more significant contribution of this network in cognitive prediction.

We next investigated which functional networks contained boundaries most strongly linked to cognitive performance, recognizing that boundary-behavior associations may not be uniformly distributed across networks. For each of the ten functional networks, we identified boundaries showing significant positive or negative associations with ELC scores and quantified their relative contribution to prediction. Specifically, we computed the mean fraction of positively and negatively associated boundaries in each network, defined as the proportion of selected boundaries (positive or negative) normalized by the total number of boundaries within that network (Fig. 5**b**). Among the most contributive networks, positively associated boundaries were predominantly located in the peripheral visual, central visual, and dorsal attention networks, whereas negatively associated boundaries were mainly concentrated within the posterior default mode network.

To further examine the importance of each network, we employed an iterative leave-one-network-out approach, where cognitive scores were predicted using all but one removed network, and the performances were averaged across cross-validations. After removing a network, a decrease in prediction accuracy indicates that the excluded network made a substantial contribution in cognitive prediction. As shown in Fig. 5**c**, prediction accuracy dropped most when the visual and dorsal attention networks were removed, underscoring their key roles in supporting cognitive performance.

## 3 Discussion

In this study, we explored the capability of local gradients of functional connectivity to capture individualized characteristics by applying them to fingerprinting and cognitive prediction across different age ranges and functional states. We found that the local gradient map (LGM) enables highly stable and accurate individual identification, consistently outperforming conventional connectome-based fingerprints. Our analysis demonstrated that the most contributive vertices for identification were primarily located in the visual, posterior default mode, and posterior frontoparietal networks. Meanwhile, cognitive prediction analyses revealed that regions associated with cognition, as identified through LGM, were predominantly located in the visual and dorsal attention networks. These results suggest that LGMs capture fine-grained, spatially localized functional patterns with region-specific robustness during dynamic early brain development, which are overlooked by conventional connectome-based fingerprints, thereby offering a novel perspective on individualized functional characteristics and their relationship with behavioral phenotypes during infancy.

### 3.1 Local gradient map-based functional fingerprints are robust, age-invariant and state-invariant

We found that local gradients of FC exhibit distinct individual fingerprinting characteristics during infancy, achieving significantly better accuracy and stability compared to conventional connectome-based fingerprints, whether applied to age-varied or state-varied groups. Despite the dynamic nature of infant brain development, we achieved an average identification rate exceeding 98% with an average session gap even around 200 days, firmly demonstrating the existence of functional fingerprints in early developing brains. This marks a significant improvement over previous studies, which reported identification rates of 11% in the perinatal period (Ciarrusta et al., 2022), 62% during infancy (Hu et al., 2022), and 42% in older children (Vanderwal et al., 2021) using resting-state functional connectomes. For state-varied comparisons (awake vs. asleep), LGM achieved a perfect 100% identification rate, whereas the connectome-based method reached only 66.7% accuracy. Previous research has conducted connectome-based fingerprinting across resting-state and task-based fMRI scans and achieved satisfactory results in late childhood (Kaufmann et al., 2017), suggesting that connectome-based fingerprints exhibit stability across different states at older ages. However, their poor performance in infancy, a period of extremely rapid neurodevelopment, highlights the limitations of connectome-based fingerprints in capturing individualized features across ages and different physiological states. In contrast, LGM demonstrates far greater stability and discriminability, underscoring its superiority for developing brain fingerprinting.

While LGM demonstrates considerable reliability and robustness during early infancy, multiple factors likely contribute to the capability to capture the individualized characteristics. We examined how different combinations of patch number and patch size in the LCM computation affect the capacity of LGMs to capture functional fingerprinting. Across all datasets and developmental stages, finer spatial resolutions consistently yielded better identification performance than coarser ones, indicating that fine-grained LGM features better preserve individual-specific functional details. Another factor is the influence of phase encoding direction (PED) in resting-state fMRI acquisitions, as varying PEDs can introduce distinct patterns of signal distortion and loss (Mori et al., 2018). Notably, our LGM-based functional fingerprint maintained high identification rates with mixed PED scans compared to connectome-based approaches (Fig. 3d), suggesting their robustness to signal distortions introduced by varying PEDs.

Understanding how local gradients of FC capture unique and stable characteristics during brain development is challenging. The local gradient of FC captures sharp spatial changes in RSFC profiles, thereby intrinsically representing transitions between functional parcels, many of which are likely preserved across development. This representation differs fundamentally from conventional connectome-based approaches, which characterize the strength and pattern of functional connectivity between regions. Importantly, two individuals with similar overall connectivity patterns may nevertheless differ substantially in the spatial locations of these functional transitions. LGMs are therefore sensitive to the precise topographic organization of functional boundaries, which has been shown to be highly reproducible within individuals yet variable across individuals (Braga and Buckner, 2017, Gordon et al., 2017, Laumann et al., 2015), rather than primarily reflecting the magnitude or configuration of interregional functional connectivity. The higher identification accuracy of LGMs observed above provides empirical support for this interpretation. Prior research has shown that functional brain networks undergo progressive subdivision, characterized by evolving intra- and inter-modular connectivity patterns, indicating enhanced functional segregation and integration during development (Wen et al., 2019). This developmental trajectory implies continuous modification of functional networks throughout infancy. The constraints imposed by predefined parcellations in connectome-based fingerprinting methods may inadequately account for these dynamical developmental changes, partially explaining the reduced identifiability observed in longitudinal studies (Hu et al., 2022, Ciarrusta et al., 2022). Local gradients, on the other hand, have been demonstrated to reflect distinct aspects of architectonic boundaries and exhibit heightened sensitivity to specific biological demarcations, correlating meaningfully with behavioral outcomes (Gao et al., 2015b, Messé, 2020). This highlights the necessity of exploring individualized functional characteristics at a local level during infancy without reliance on parcellations. Hence, the local gradients-based functional fingerprints are likely attributable to their capabilities to capture fine-grained localized spatial information that exhibits region-variable stability during development and typically remains undetectable at global scales of analysis in conventional methods.

### 3.2 Network-specific Contributions to Individual Identifiability

Several networks exhibited consistently high identifiability and uniqueness, including the central visual, posterior frontoparietal, and posterior default mode networks (Fig. 4). The high performance of association networks (frontoparietal and default mode) aligns with previous reports showing that these regions exhibit greater interindividual variability in connectivity profiles and contribute substantially to functional identifiability (Miranda-Dominguez et al., 2014, Hill et al., 2010, Wang et al., 2021a).

Beyond these expected findings, the central visual network exhibited strong uniqueness and identifiability, which was unexpected given its early maturation and structural stability. While the theoretical basis of this observation remains to be fully clarified, prior evidence provides indirect support for such a pattern. Functional MRI studies have reported measurable interindividual variability in visual networks (Liu et al., 2018, Gao et al., 2014), alongside test-retest reproducibility of visual network across sessions (Wang et al., 2021b). These dual properties, between-subject variability and within-subject stability, may be effectively captured by LGM, enabling fine-grained local gradients to retain sufficient individual-specific variation for reliable identification.

Beyond these top-performing networks, our results revealed that the majority of functional networks, e.g., superior temporal, dorsal attention, peripheral visual, etc., also contributed to high uniqueness and differential power, indicating a distributed contribution across the cortex. This widespread pattern of identifiability contrasts with prior connectome-based findings. Prior connectome-based studies have shown that identification is typically driven by a small subset of association networks, such as the frontoparietal networks (Emily et al., 2015, Wang et al., 2021a, Mueller et al., 2013). In contrast, the distributed contribution of local gradients indicates that individual uniqueness is not confined to a limited set of high-variability regions but instead emerges from fine-scale connectivity transitions spanning the entire cortex.

### 3.3 Association between local gradients of functional connectivity and cognition

We also observed a modest association between LGM and cognitive behaviors, consistent with existing studies indicating the relationship between individual uniqueness and cognitive behaviors (Emily et al., 2015, Horien et al., 2019, Kaufmann et al., 2017, Hu et al., 2022, Cole et al., 2012). Among the ten functional networks (Wang et al., 2023a), our analyses identified the visual network, dorsal attention network, and anterior frontoparietal network as the networks contributing most to this association. The visual network is relatively mature with adult-like topology after birth (Gao et al., 2015b, Doria et al., 2010, Smyser and Neil, 2015), with significant increases in functional connectivity strength and heterogeneity during early development (Cao et al., 2017). These developmental patterns, which are closely linked to brain activity in cognitive behavior, can be well captured by LGM.

Interestingly, previous research comparing LGM- and connectome-based approaches for cognitive prediction suggested that local gradient methodologies might lack behaviorally relevant information (Kong et al., 2023). A likely explanation is that the earlier study computed global similarity between entire LGMs using Pearson correlation, which yields a single similarity score and overlooks spatially localized information. Such global correlation may obscure the fine-scale localized variations that are important to interpreting LGMs. In contrast, our approach evaluates correlation between cognition and each boundary, preserving fine-scale spatial information and capturing localized variations. In addition, differences in predictive modeling strategies may also have contributed to the discrepancy. The earlier study applied a basic kernel regression model using the full set of features without feature selection or dimensionality reduction, likely introducing irrelevant or noisy predictors and reducing overall performance (Emily et al., 2015, Hu et al., 2022). Collectively, these considerations suggest that the behavioral relevance of local gradients may be best understood from a spatially localized perspective, as LGMs intrinsically capture abrupt transitions in functional connectivity that are not preserved in global representations.

These findings should nonetheless be interpreted with caution. The cognitive analysis was based on a relatively modest behavioral sample (116 subjects, 197 scans) and was evaluated using repeated subject-level cross-validation, rather than in an independent replication cohort. The observed association, while significant (*r* = 0.294, 95% CI [0.188, 0.423], *p* < 0.001) was modest in magnitude. We therefore regard these results as evidence that local gradients carry behaviorally relevant information and note that replication in larger, independent infant samples will be needed to confirm their generalizability.

### 3.4 Potential developmental basis of individual-specific LGMs

The individual specificity captured by LGMs may partly reflect an early-established spatial scaffold of cortical functional organization. Cortical arealization begins prenatally and is shaped by spatially patterned molecular and transcriptional programs, suggesting that the relative organization of neighboring cortical territories is constrained well before mature functional networks are fully established (Bhaduri et al., 2021, Pletikos et al., 2014). Consistent with this view, organized functional architecture and interindividual variability in functional connectivity are already detectable during the prenatal and neonatal periods (Gao et al., 2014, Xu et al., 2019). At the same time, functional connectivity strength continues to change substantially throughout late gestation and infancy, with primary sensory and motor systems maturing earlier than higher-order association systems (Cao et al., 2017, Eyre et al., 2021). These later changes likely reflect multiple interacting developmental processes, including myelination, synaptic refinement, and maturation of structural and functional connectivity.

Because an LGM captures local spatial transition between neighboring functional connectivity profiles rather than the absolute strength of individual connections, an individual-specific spatial arrangement of these transitions may remain informative even as the underlying connectivity continues to mature. The high longitudinal identifiability of LGMs reflects, at least in part, a relatively stable individualized spatial scaffold that emerges early during development. However, this does not imply that functional boundaries are genetically fixed or developmentally invariant. Rather, their magnitudes may continue to evolve in a region-specific manner, while preserving sufficient individual-specific spatial organization to support individual identification across development. LGMs should therefore be interpreted as systems-level functional phenotypes rather than direct measures of genetic patterning, myelination, synaptic organization, or any other single biological process.

### 3.5 Additional Considerations

Our findings should be interpreted in light of several limitations. First, we focused on static functional connectivity and its derived local gradient-based functional fingerprint to investigate fine-grained, robust individualized patterns under development. Exploring whether dynamic functional connectivity and its derived measures (Liu et al., 2018) can better characterize individual uniqueness would further improve our understanding of the inherent traits and ongoing development. Second, all experiments were conducted during infancy featuring extremely dynamic development. Given the strong performance of local gradient-based functional fingerprints, it would be interesting to test their reproductivity in other age groups, e.g., older children, adolescence, and so on. Third, we only used typically developing infants in our study. We can also apply our method on subjects with or at-risk for neurodevelopmental disorders to advance our understanding of whether and how these will influence the local gradient-based functional fingerprints.

In addition to these limitations, computational scalability is an important practical consideration. FC-gradient estimation is the most computationally intensive step, but it is performed only once per scan and the resulting gradient maps are stored for reuse. At 40,962 vertices, FC-gradient estimation required approximately 8 h per scan with a peak memory usage of 64 GB, whereas local correlation-map generation required only 0.42 s per scan at 40,962 patches. Importantly, comparable identification performance was consistently achieved across the three surface resolutions under all evaluated parameter settings. This indicates that lower-resolution FC gradients provide a computationally efficient alternative for large-scale analyses, reducing the runtime and peak memory to approximately 1 h and 8 GB at 10,242 vertices, and 10 min and 1 GB at 2562 vertices. In addition, because scans are processed independently, FC-gradient estimation can be readily parallelized across participants, further improving scalability for large-scale studies and clinical research datasets.

## 4 Conclusion

In summary, our findings indicate that local gradients of functional connectivity capture individualized fine-grained, spatially localized features that offer a novel perspective for understanding functional individuality across development. Complementing previous work that focuses on global connectome-based approaches, our findings highlight the importance of fine-scale local transitions in shaping individual functional architecture. This localized perspective is particularly valuable during early brain development, when rapid and heterogeneous developmental trajectories challenge conventional connectome-based approaches. By establishing the behavioral relevance of local gradients, our study contributes to the growing shift toward individualized functional mapping and supports the potential use of such localized patterns for characterizing cognitive traits and, pending validation in clinical populations, for identifying early deviations associated with neurodevelopmental disorders, which represents an important direction for future research.

## 5 Methods

### 5.1 Participants and data acquisition

Subjects in this study are from the public UNC/UMN Baby Connectome Project (BCP) (Gilmore et al., 2018). Aiming to study normal early brain development, all infants recruited in BCP were born at the gestational age of 37–42 weeks and were free of any major pregnancy and delivery complications.

All MRIs were acquired during infants’ natural sleep using 3 T Siemens Prisma MRI scanners with Siemens 32-channel head coils. T1-weighted images (208 sagittal slices) were obtained by using the three-dimensional magnetization-prepared rapid gradient echo sequence: repetition time (TR), echo time (TE), and inversion time (TI) = 2400, 2.24, and 1600 ms, respectively; flip angle = 8°, and resolution = 0.8 × 0.8 × 0.8 mm^3^. T2-weighted images (208 sagittal slices) with turbo spin-echo sequences (turbo factor = 314, echo train length = 1166 ms): TR, TE = 3200, 564 ms, respectively; and resolution = 0.8 × 0.8 × 0.8 mm^3^ using a variable flip angle. All structural MRI scans were assessed visually for excessive motion, insufficient coverage, and/or ghosting to ensure sufficient image quality for processing. For the same cohort, resting-state functional MRI (rs-fMRI) scans were acquired using a blood oxygenation level-dependent (BOLD) contrast-sensitive gradient echo-planar sequence: TR = 800 ms, TE = 37 ms, flip angle = 80°, field of view = 208 × 208 mm, 72 axial slices per volume, resolution = 2 × 2 × 2 mm^3^, and total volumes = 420. fMRI scans include both anterior to posterior (AP) scans and posterior to anterior (PA) scans, which are two opposite phase encoding directions (PEDs) for better correction of geometric distortions. In this study, we tested our model in three groups: AP, PA, and average, where the average group combines the scans acquired from the same subject at the same age (averaging AP and PA scans).

**Inclusion and exclusion criteria.** All data were preprocessed with iBEAT V2.0 Cloud (Wang et al., 2023b). Throughout, a scan denotes a single imaging timepoint (one visit, anchored on the structural acquisition), and a run denotes an individual rs-fMRI acquisition obtained within a scan. Each scan required a usable T1w or T2w image for tissue segmentation and cortical surface reconstruction to provide a surface representation onto which the functional time series were resampled. Reconstructed surfaces were subsequently reviewed by expert raters, and a scan was retained only if its reconstruction was free of visible anatomical and topological errors. Of the 306 enrolled subjects (775 scans), 249 subjects (546 scans) passed this review, 211 subjects (493 scans) fell within the age range of birth to 874 days, and 132 subjects (342 scans, 936 rs-fMRI runs) had rs-fMRI runs acquired in both AP (Anterior-to-Posterior) and PA (Posterior-to-Anterior) phase-encoding directions. These 132 subjects constituted the analytic pool for the identification and cognitive analyses.

The identification analyses included 103 subjects with at least two scans, yielding 303 scans acquired between 16 and 874 days after birth. Cognitive ability was assessed using the Early Learning Composite (ELC) scores (M Mullen et al., 1995, Yitzhak et al., 2015). For the cognitive analyses, scans were included when a valid ELC score was available within one month of imaging, resulting in 116 subjects and 197 scans. Of the 132 subjects in the analytic pool, 87 were included in both analyses, 16 were included only in the identification analyses because they had no eligible ELC-matched scan, and 29 were included only in the cognitive analyses because they did not meet the longitudinal scan requirement for identification. Fig. 1**a** summarizes the sample selection and overlap between analyses.

In addition, 21 older BCP subjects (3–6 years), who were not included in the samples described above, underwent paired asleep and awake acquisitions on the same day and were processed using the same quality-control procedure, enabling comparison of functional MRI across states.

### 5.2 Data processing

All structural and functional MRIs were processed following state-of-the-art infant-tailored pipelines (Zhang et al., 2019a, Li et al., 2019). All reconstructed cortical surfaces were first registered onto the UNC 4D infant cortical surface atlas (Wu et al., 2019, Li et al., 2015) based on cortical geometric features. The functional time series at each vertex on the resampled middle cortical surface was then extracted. For each cortical surface, the local gradient map (LGM) of functional connectivity was then computed according to (Gordon et al., 2016, Wang et al., 2023a) and further resampled with 40,962 vertices (Fig. 1**b**).

**Structural MRI processing.** All T1- and T2-weighted magnetic resonance images underwent processing using an infant-specific pipeline iBEAT V2 (Wang et al., 2023b) that has been extensively validated in many infant studies. The processing protocol encompasses multiple sequential steps. First, T2-weighted images were rigidly aligned onto their corresponding T1-weighted images (Smith et al., 2004, Jenkinson et al., 2002, Woolrich et al., 2009). Subsequently, skull stripping was performed using a deep learning-based method, followed by manual refinement to ensure complete removal of skull and dura matter (Zhang et al., 2019b). The cerebellum and brain stem were then removed through atlas-based registration, followed by correction of intensity inhomogeneity using the N3 method (Sled et al., 1998). Brain images were segmented into white matter, gray matter, and cerebrospinal fluid utilizing an infant-dedicated deep learning-based approach. Finally, each brain was separated into left and right hemispheres with subsequent filling of non-cortical structures.

**Functional MRI processing.** fMRI data were preprocessed using an infant-specific functional pipeline with the following steps (Zhang et al., 2019a, Zhou and Zhang, 2019): correction of head motion and spatial distortion using FSL (Smith et al., 2004), registration of rs-fMRI scans onto the corresponding T1w MRI using a boundary-based registration approach (Greve and Fischl, 2009, Griffanti et al., 2014), concatenation of transformation and deformation fields to resample fMRI data through a one-time resampling strategy, and performing a conservative high-pass filter with a sigma of 1000 s to remove the linear trends. After that, the individual independent component analysis was conducted to decompose each preprocessed rs-fMRI scan into 150 components using MELODIC (Beckmann and Smith, 2004) in FSL and then a deep learning-based noise-related component identification algorithm was used to remove non-signal components (Kam et al., 2019).

**Cortical surface reconstruction and mapping.** Following tissue segmentation, inner, middle, and outer cortical surfaces were reconstructed using topology-preserving deformable surface methods (Li et al., 2012). After surface reconstruction, the inner cortical surface, which has vertex-to-vertex correspondences with the middle and outer cortical surfaces, was further smoothed, inflated, and mapped onto a standard sphere (Fischl et al., 1999). All spherical cortical surfaces were aligned onto UNC 4D infant cortical surface atlases with Spherical Demons (Thomas Yeo et al., 2009) and further mapped onto the HCP 164k fs_LR space. All surfaces were ultimately resampled to 32k fs_LR space (Glasser et al., 2013) to establish vertex-to-vertex correspondence across subjects and ages. Finally, fMRI time courses were resampled onto the middle cortical surface and spatially smoothed for subsequent analysis.

**Computation of local gradient map (LGM) of functional connectivity.** We computed the LGM of functional connectivity, which indicates sharp transitions in resting-state functional connectivity (RSFC) and inherently delineates boundaries between functional parcels. This approach has been extensively validated for generating meaningful fMRI-based cortical parcellations in adult populations (Gordon et al., 2016, Schaefer et al., 2018). We processed anterior-posterior (AP) and posterior-anterior (PA) scans independently, integrating them only in the final step. For each scan, we constructed a functional connectivity matrix by computing pair-wise correlations of BOLD signals between all cortical vertices, yielding a 32k × 64k RSFC matrix per hemisphere, with each row representing a vertex-specific RSFC map across two hemispheres. The FC matrices then underwent Fisher's r-to-z transformation, normalizing features across vertices to ensure comparative scaling. Subsequently, we generated a second-order correlation matrix (RSFC-2nd) sized 32k × 32k per hemisphere by correlating z-transformed RSFC maps across all cortical vertices within the same hemisphere. While primary RSFC maps exhibit gradual transitions, these second-order correlation maps demonstrate more pronounced transitions, thereby facilitating the identification of functional boundaries between adjacent areas (Wig et al., 2014). Following the methodology in (Glasser et al., 2016), we calculated the gradient of FC on the RSFC-2nd, producing a 32k × 32k gradient matrix per hemisphere. Utilizing watershed-based boundary detection protocols (Gordon et al., 2016), we generate 32k binary boundary maps per hemisphere from the gradient matrix. The local gradient map is defined as the mean of the 32k binary boundary maps. For scan sessions containing both AP and PA acquisitions, the local gradient maps from AP and PA scans are averaged. Finally, the local gradient maps were further mapped to the fsaverage space for functional fingerprinting analysis.

### 5.3 Local-level Local gradient map-based functional fingerprinting (LGM-Local)

For functional fingerprinting, we constructed a base set, $B=\{G_{j}^{b},j=1,2,\ldots ,N\}$, and a target set, $T=\{G_{i}^{t},i=1,2,\ldots ,N\}$, where N denotes the number of subjects, and $G_{i}^{t}$ and $G_{i}^{b}$ are LGMs at session t and session b, respectively. In the process of functional fingerprinting, each individual (to-be-identified) $G_{i}^{t}$ was selected from the target set T, and the local correlation map (LCM) between $G_{i}^{t}$ and each $G_{i}^{b}$ in the database set B, denoted as $C_{\mathrm{ij}}$, was computed to identify this individual. We performed this procedure in both directions: first with session S1 serving as the target set T and session S2 as the base set B (S1→S2), and then with the roles reversed, session S2 as the target set T and session S1 as the base set B (S2→S1).

**Computation of local correlation maps (LCMs).** For each sampled vertex v, we first extracted its local surface patches within *r*-ring neighborhood, $P_{i}^{t}\left(v\right),\;$for representing its local fine-grained functional pattern. The LCM was computed between corresponding surface patches of two LGMs $G_{i}^{t}$ and $G_{j}^{b}$, and this procedure is defined as: $C_{\mathrm{ij}}(v)=Corr(P_{i}^{t}(v),\;P_{j}^{b}(v))$, where Corr is the Pearson’s correlation coefficient (Fig. 6).

**Fig. 6:**
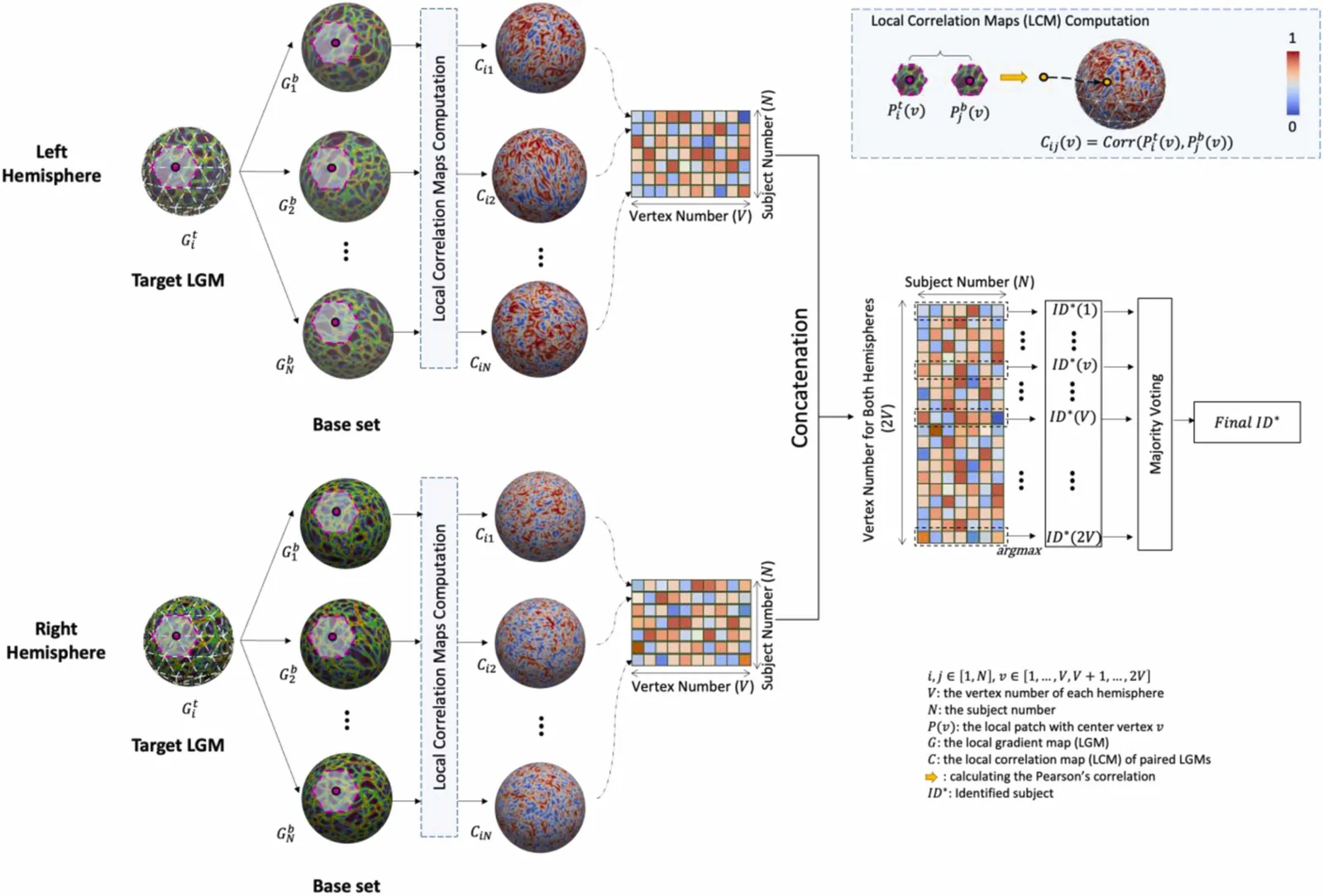
The process of local-level local gradient map-based functional fingerprinting (LGM-local). For a query subject’s LGMs of left and right hemispheres from the target set, we computed local correlation maps (LCMs) against corresponding hemispheres in the base set. Then the left and right LCMs were combined into a similarity matrix. The identity of the target LGM is determined by majority voting across vertex-wise predictions.

For the main identification analyses, LCMs were computed using *V* ∈ {40,962, 10,242, 2562} uniformly sampled vertices on the standard fsaverage sphere, with patch size based on *r*-ring neighborhood (*r* ∈ {1, 2, 3, 4, 5, 6}). To separate parameter selection from performance estimation, we evaluated LGM using subject-level nested cross-validation, applied independently to Datasets I–III. The 103 subjects were partitioned into 10 outer folds. For each outer fold, the remaining nine folds served as the training set and were further divided into five inner folds. Candidate configurations, consisting of all combinations of patch number and patch size, were compared using only the inner-fold identification accuracy. The best-performing configuration was then evaluated once on the held-out outer-fold subjects, who took no part in parameter selection. Identification was performed with the held-out subjects as queries against the full 103-subject gallery (chance ≈ 1/103). Held-out accuracies from the ten outer folds were pooled and are reported as mean ± SD. Statistical significance was assessed using a permutation test in which subject-identity labels within each held-out fold were randomly shuffled and identification accuracy recomputed 5000 times to generate the null distribution.

To evaluate robustness under single phase-encoding scans, we additionally implemented a deformable patch strategy. This strategy slightly deforms local surface patches to compensate for residual registration errors by allowing both translation and rotation of the patches. Specifically, each patch was translated in six directions on the tangent plane and rotated by ±60° about its center, generating six translation-based and two rotation-based correlation values, respectively. For each vertex, the maximum correlation among the original, translated, and rotated patches was retained. The deformable parameters consisted of ±60° rotations and translations along the six natural directions of the hexagonal neighborhood defined by the icosahedral tessellation. This strategy was used only as a robustness analysis for single phase-encoding scans (Results, Fig. 3**c**) and was not included in the parameter search or the main identification pipeline.

The configuration selected by the nested cross-validation was fixed for all subsequent analyses. The full-sample identification accuracy is reported in the Results only as a descriptive summary and is not used as the primary estimate of generalization performance.

Majority Voting-based individual identification. For each hemisphere of each target LGM, we first computed its LCM with each LGM in the base set, thus generating N LCMs. Subsequently, we concatenated LCMs from both hemispheres and constructed a similarity matrix, denoted as $S\in {\mathbb{R}}^{N\times 2V}$, where each column represents a cortical vertex and each row represents a subject from the base set. Specifically, the first V columns correspond to the left hemisphere, while the remaining V columns represent the right hemisphere.

To obtain the identity of the given subject based on the similarity matrix, we leveraged a vertex-wise identification method. For each cortical vertex v∈{1,…,V,V+1,…,2V}, we assigned the identity of the subject from the base set who showed the highest similarity at that vertex:

$$Identity_{v}(G_{i}^{t})=argmax_{j\in \{1,2,...,N\}}S(j,v)$$

This procedure yields a predicted identity at each vertex, denoted as $I\mathrm{dentit}y_{v}$, for target LGM $G_{i}^{t}$. We then applied a majority voting scheme across all sampled vertices to determine the final identity. Specifically, we counted how frequently each identity appeared among the vertex-wise predictions and selected the most frequent one:

$$Identity\left(G_{i}^{t}\right)={argmax}_{j\in \left\{1,2,\ldots ,N\right\}}\sum_{v=1}^{2V}\sigma \left({Identity}_{v}\left(G_{i}^{t}\right)=j\right),$$

where σ is the indicator function, which returns 1 if the prediction at vertex v equals identity j, and 0 otherwise. This summation effectively counts the number of vertices supporting each identity, enabling selection of the most frequently predicted subject.

This majority-voting framework was motivated by the spatially distributed and heterogeneous nature of subject-specific information in FC gradients. Individual cortical locations may differ in their discriminative information and may be affected by residual registration error, transient head motion, or low local signal-to-noise ratio. Rather than reducing the entire cortical surface to a single similarity value, each sampled vertex contributes a local identity estimate based on its surrounding patch, and the final identity is obtained by aggregating these local decisions. The resulting decision is thus less sensitive to unreliable information from a subset of cortical locations. The aggregation rule was fixed and contained no tunable parameters. All sampled vertices contributed equally, with no vertex selection, weighting, regional masking, or vote-count threshold, and the voting rule was not varied across analyses. Only the number of sampled vertices (*V*), and patch size (*r*), were selected, exclusively within the inner folds of the nested cross-validation procedure described above. Thus, the voting step did not introduce an additional parameter-selection process that could inflate the estimated identification performance.

### 5.4 Global-level Local gradient map-based functional fingerprinting (LGM-Global)

Unlike LGM-Local, this approach predicts identity from the global similarity of whole-cortex LGM, which is similar to connectome-based identification. Similarly, for the target subject, we first concatenate the LGMs of left- and right-hemisphere to form a whole-cortex representation and do the same for LGMs in base sets. We then compute the Pearson’s correlation between target and base LGMs, and assign the identity with the highest similarity in the base set: ${Identity(G}_{i}^{t})=argmax_{j}Corr\left(G_{i}^{t},\;G_{j}^{b}\right)$, where Corr is the Pearson’s correlation coefficient.

### 5.5 Functional connectome-based individual identification

To benchmark LGM-based identification against conventional connectome-based approaches, we compared performance using two parcellation schemes that differ in both spatial resolution and population specificity. The first was a 360-ROI parcellation (Glasser et al., 2016), a widely used, publicly available atlas that enables comparison with prior connectome-based fingerprinting studies employing comparable standard parcellations (Hu et al., 2022). The second was an 864-ROI parcellation specifically developed for the infant cortex (Wang et al., 2023a), offering substantially finer spatial resolution while more closely reflecting infant-specific anatomical and functional organization compared to adult-derived atlases. Together, these two parcellations provide complementary benchmarks. The 360-ROI atlas serves as a standard connectome-based reference that facilitates comparison with prior fingerprinting studies, whereas the 864-ROI infant-specific atlas provides a more stringent benchmark to test whether any advantage of LGM could be attributed to either insufficient parcellation granularity or a mismatch between adult-derived atlases and infant cortical organization.

The connectome-based individual identification was performed across paired scans consisting of one target set, $T=\{X_{i}^{t},i=1,2,\ldots ,N\}$, and one base set, $B=\{X_{j}^{b},j=1,2,\ldots ,N\}$, where $X_{i}^{t}$ and $X_{j}^{b}$ are all flattened vectors of the upper triangle of the whole-brain connectivity matrix, the superscripts b and t denote subject from base or target set, and the subscripts i and j denote the subject index. In the process of identification, one target vector $X_{i}^{t}$ was selected from the target set T, and Pearson’s correlation coefficient was used to measure the similarity between $X_{i}^{t}$ and each $X_{j}^{b}$ in the base set B. The identity of $X_{i}^{t}$ is assigned to the subject in the base set with the highest similarity, i.e., ${Identity(X}_{i}^{t})=argmax_{j}Corr(X_{i}^{t},\;X_{j}^{b}).\;$Moreover, previous study found that proper selection of edges can improve the identification accuracy in infants (Hu et al., 2022), so we integrated feature selection-based identification via estimating the differential capability of an edge across subjects. Because feature selection was involved in the identification process, we designed an identification-specific 10-fold cross-validation and repeated it 20 times for the infant identification analysis.

### 5.6 Quantifying vertex-wise contributions to identification

To ascertain vertex-wise contribution to the individual identification process, we used two measures: uniqueness (Tian et al., 2021, Mueller et al., 2013) and differential power (DP) (Emily et al., 2015, Wang et al., 2021a). Vertices with high uniqueness indicate their functional patterns are consistent within the same individual across age, but varied widely across individuals, while those with high DP values are thought to make large contributions to individual identification.

**Uniqueness.** To identify the specific loci that manifest individual uniqueness, we adopted the measure of uniqueness (Tian et al., 2021, Mueller et al., 2013), which quantifies the spatial distribution of inter-subject variability and provides important insights into individual differences. The uniqueness is calculated based on the average intra-individual local correlation maps $C_{\mathrm{intra}}$ and average inter-individual local correlation maps $C_{\mathrm{inter}}$. For each dataset, the average intra-individual correlation map was derived from the same individual from different ages, $C_{\mathrm{intra}}=\frac{1}{N}\sum_{i,i\in \left\{1,2,\ldots ,N\right\}}C_{\mathrm{ii}}$. The average inter-individual correlation map was derived from different pairs of individuals, $C_{\mathrm{inter}}=\frac{1}{N\times (N-1)}\sum_{i,j\in \left\{1,2,\ldots ,N\right\},i\neq j}C_{\mathrm{ij}}$. To estimate the uniqueness map, the intra-individual correlation map $C_{\mathrm{intra}}$ and inter-individual correlation map $C_{\mathrm{inter}}$ were subtracted from a vector of all ones, l, namely intra-individual variance and inter-individual variance. Then the intra-individual variance was regressed out from inter-individual variance using ordinary least squares regression. The residual map between regressed intra-individual variance and inter-individual variance was considered as the uniqueness map (U). This process was as follows: $U=\left(l-C_{inter}\right)-\alpha \left(l-C_{intra}\right)-\beta$, where α and β are parameters determined via ordinary least-squares. Hence, the uniqueness map U would exhibit high values for vertices that demonstrate substantial variability among individuals and are consistent across age within the same individual. The uniqueness maps derived from different datasets were first normalized using z-scores and then averaged for subsequent analysis.

**Differential power.** Given a vertex v in LCM, if the vertex positively contributes to the identification of the individual, it should satisfy $C_{\mathrm{ii}}\left(v\right)>C_{\mathrm{ij}}(v)$ for the identification process from session base to session target and $C_{\mathrm{ii}}\left(v\right)>C_{\mathrm{ji}}(v)$ from session target to session base. The DP for a vertex at the individual level is calculated as: $DP_{i}\left(v\right)=-\ln\frac{\left(\left|C_{\mathrm{ii}}\left(v\right)<C_{\mathrm{ij}}\left(v\right)\right|+\left|C_{\mathrm{ii}}\left(v\right)<C_{\mathrm{ji}}\left(v\right)\right|\right)}{2(N-1)}$, where $\left|C_{\mathrm{ii}}\left(v\right)<C_{\mathrm{ij}}\left(v\right)\right|$ and $\left|C_{\mathrm{ii}}\left(v\right)<C_{\mathrm{ji}}\left(v\right)\right|$ denote the total number of times that $C_{\mathrm{ii}}\left(v\right)<C_{\mathrm{ij}}(v)$ and $C_{\mathrm{ii}}\left(v\right)<C_{\mathrm{ji}}(v)$ across all other individual *j*, respectively. The vertex-wise DP for the population is calculated as: $\mathrm{DP}\left(v\right)=\frac{\sum_{i}{\mathrm{DP}}_{i}(v)}{N}$. A higher DP value indicates a greater contribution of that vertex to individual identification.

### 5.7 Cognitive score prediction

To investigate the association between individual functional fingerprints and infant cognitive development, we evaluated the predictive capacity of local gradient-based functional fingerprints for cognitive performance. Cognitive ability was assessed using the Early Learning Composite (ELC) score derived from the Mullen Early Learning Scale Assessment (M Mullen et al., 1995, Yitzhak et al., 2015), which aggregates scores from four domains: Fine Motor (FM), Visual Reception (VR), Receptive Language (RL), and Expressive Language (EL).

A random forest (RF) model (Kesler et al., 2017, Wlaszczyk et al., 2019) was used to predict ELC scores from the selected LGM-derived boundary features. Model performance was evaluated using 10 repeated iterations of subject-level 10-fold cross-validation. All longitudinal scans from the same participant were assigned exclusively to either the training or testing set within each fold, thereby preventing subject-level information leakage. Feature selection was performed independently within each training fold, with the corresponding test data completely excluded from feature selection. The RF model used 20 ensemble trees and a minimum leaf size of 5, following settings used in a prior study (Hu et al., 2022) and these parameters were kept fixed throughout the cross-validation without data-driven hyperparameter tuning. For each repetition, ELC scores of the held-out subjects were predicted using the trained RF model. After all 10 repetitions were completed, the predicted values obtained across repetitions were averaged for each scan to obtain the final predicted scores. Predictive performance was assessed using Pearson’s correlation coefficient between the predicted and observed ELC scores.

Feature construction followed the feature-selection framework commonly used in connectome-based prediction (Emily et al., 2015), but was adapted to the natural units of our representation. Whereas connectome-based methods select connectivity edges, our local gradient maps are naturally characterized by functional boundaries. This choice is further supported by prior evidence linking cortical boundaries to cognition (Gao et al., 2015b, Messé, 2020). Candidate boundaries were defined as the interfaces between adjacent cortical ROIs in a group-level, local-gradient-derived infant functional parcellation (Wang et al., 2023a). The group-level parcellation was used here to provide dimensionality reduction and a consistent feature space across subjects, both of which are necessary for cognitive prediction using machine learning. Specifically, it defines a common set of anatomically corresponding boundary sets at which LGM features can be sampled and compared across individuals. Importantly, the parcellation is used solely to define these shared sampling locations; the feature value at each boundary is derived from each infant's own individualized LGM map and therefore remains subject-specific. To improve robustness to minor spatial misalignment, each boundary was dilated by one vertex on both sides. For each boundary, the mean local-gradient magnitude across the included vertices was calculated from the subject's own LGM and used as the corresponding feature for prediction.

The use of boundaries as the feature representation was a priori, theory-motivated choice, whereas the specific boundaries included in the model were selected within each training fold only, with no involvement of the test data at any stage of selection. Boundaries showing correlations with ELC at p < 0.1 in the training data were retained and divided into positive and negative feature sets, with no test data used during feature selection. For each subject, two summary statistics, positive strength and negative strength, were derived by summing all correlation values across the positively and negatively associated boundaries, respectively, defined as:

$$S_{\mathrm{Positive}}=\sum_{k}r_{k}g_{k}m_{k}^{+},$$

$$S_{\mathrm{Negative}}=\sum_{k}{r_{k}g_{k}m}_{k}^{-},$$

where $r_{k}$ is the Pearson correlation coefficient of boundaries and cognition, $g_{k}$ is the local gradient value of boundary k, and $m_{k}^{+}=1$, $m_{k}^{-}=0$ where $r_{k}>0$, otherwise $m_{k}^{+}=0$, $m_{k}^{-}=1$. Moreover, a random forest (RF) model was adopted to capture the relationship between the difference of two single-subject summary statistics ($S_{\mathrm{Positive}}-S_{\mathrm{Negative}}$) and the dependent variable, ELC. The RF framework was chosen because its inherent bootstrapping strategy, combined with the use of summarized boundary-level features, effectively mitigates the issue of limited sample size in ELC prediction. In the RF analysis, the number of ensemble trees was set to 20 and the minimum leaf size to 5, following settings used in prior study (Hu et al., 2022). During testing, the fitted RF model was applied to predict the ELC scores directly.

To compute the differential contributions of functional networks to cognitive prediction, we computed mean fraction of contributing boundaries within each network, defined as the number of boundaries, normalized by the total boundaries within each network to account for the heterogeneity of network size. The contribution of the boundaries was summarized separately with the negatively and positively correlated feature set. For the boundaries located between networks, they were counted for both networks when computing contributions. Besides, we employed an iterative leave-one-network-out approach, where cognitive scores were predicted with the remaining networks, and the results were averaged across cross-validations.

## CRediT authorship contribution statement

**Xinrui Yuan:** Writing – review & editing, Writing – original draft, Validation, Software, Methodology, Investigation, Formal analysis, Data curation, Conceptualization. **Dan Hu:** Writing – review & editing, Methodology. **Jiale Cheng:** Writing – review & editing. **Zhengwang Wu:** Writing – review & editing, Data curation. **Li Wang:** Data curation. **Weili Lin:** Data curation. **Gang Li:** Writing – review & editing, Supervision, Funding acquisition, Conceptualization, Data curation.

## Code Availability

This study used openly available software and codes. The computation and analysis codes are available at https://github.com/BRAIN-Lab-UNC/LocalGradFingerprint.

## Declaration of Competing Interest

The authors declare that they have no known competing financial interests or personal relationships that could have appeared to influence the work reported in this paper.

## Data Availability

The data used in this study are publicly available from established neuroimaging repositories.
