# Supplementary material for: Unique local gradient-based functional fingerprints enable precisely identifying individual infants over ages
Source: Dev Cogn Neurosci. 2026 Oct 3;82:101828. doi: 10.1016/j.dcn.2026.101828 (PMC13677238; doi:10.1016/j.dcn.2026.101828)
Supplement: Supplementary file 1 [file mmc1.docx]

Supplementary Information for

**Unique Local Gradient-based Functional Fingerprints Enable Precisely Identifying Individual Infants Over Ages**

Xinrui Yuan *et al.*

*Corresponding author Email: gang_li@med.unc.edu

This PDF file includes:

Figure S1

**SI results**

***Developmental variation in individualized fingerprint similarity***

To further distinguish longitudinal identifiability from developmental trajectory, we examined within-subject fingerprint similarity as a function of both inter-scan age gap and developmental age. For each pair of longitudinal scans from the same subject, we calculated within-subject fingerprint similarity. To visualize the developmental trends, inter-scan age gap was divided into five bins, whereas pairwise mean scan age was divided into tertiles defining three developmental-stage groups (Early, Middle, and Late) (see **SI Methods**).

As shown in **Figure S1**, LGM similarity remained relatively high across longitudinal scans but gradually decreased with increasing inter-scan age gap. In contrast, similarity increased across the mean-age groups, indicating greater scan-to-scan similarity at later developmental stages. Thus, LGM fingerprints not only remain individually distinctive across longitudinal scans but also exhibit measurable changes in within-subject similarity with both inter-scan interval and developmental age. Connectome-based fingerprints derived from the 360- and 864-region parcellations showed broadly similar trends.

**SI Figures**

**Figure S1**. Developmental variation in within-subject fingerprint similarity. (A) Mean within-subject similarity across inter-scan age-gap bins for LGM and connectome-based fingerprints. Inter-scan age gap was divided into five bins to visualize the overall trend, with lines connecting the mean similarity within each bin. (B) Within-subject similarity as a function of the mean age of each scan pair. Mean age was divided into three developmental-stage groups based on tertiles: Early = 52–290 days, Middle = 291–484 days, and Late = 485–811 days. Lines connect the mean similarity within each group, and error bars indicate 95% confidence intervals.

**SI Methods**

***Developmental variation in within-subject fingerprint similarity***

For each infant with longitudinal scans, all pairwise combinations of scans were formed. For each pair of scans from subject $i$, vertex-wise correlation maps (local correlation maps, LCM) were computed as described in **Methods** (**Computation of local correlation maps (LCMs)**). Within-subject fingerprint similarity $\mathcal{s}$ was quantified as the mean of these vertex-wise correlations across all cortical vertices after excluding medial wall vertices: $\mathcal{s}_{i}=\frac{1}{\left| V \right|}\sum_{v\in V} \boldsymbol{C}_{i}\boldsymbol{(}v\boldsymbol{)}$, where *V* denotes the set of retained cortical vertices across both hemispheres, $\boldsymbol{C}_{i}\boldsymbol{(}v)$ is the local correlation maps at vertex *v* for subject $i$ between scans at different time points. For connectome-based fingerprints, similarity was computed analogously as the correlation between the corresponding connectivity profiles derived from the 360- and 864-region parcellations.

For visualization (**Figure S1**), inter-scan age gap was divided into five bins (≤100, 101–200, 201–300, 301–500, and >500 days), and pairwise mean scan age was divided into tertiles defining three developmental-stage groups (Early = 52–290 days, Middle = 291–484 days, Late = 485–811 days). Within each bin $\mathfrak{b}$, the group mean similarity was computed as the unweighted average across all scan pairs falling in that bin: ${\bar{\mathcal{s}}}_{\mathfrak{b}}=\left( \frac{1}{n_{\mathfrak{b}}} \right)\sum_{i\in\mathfrak{b}} \mathcal{s}_{i}$ , and the corresponding 95% confidence interval was obtained from the normal approximation: ${\bar{\mathcal{s}}}_{\mathfrak{b}}\pm1.96\cdot\frac{SD_{\mathfrak{b}}}{\sqrt{n_{\mathfrak{b}}}}$, where $SD_{\mathfrak{b}}$ is the sample standard deviation within the bin. No curve fitting or smoothing was applied. The lines in **Figure S1** connect adjacent bin means, and bins are plotted on an ordinal axis at equal spacing.
